# Biographer: web-based editing and rendering of SBGN compliant biochemical networks

**DOI:** 10.1093/bioinformatics/btt159

**Published:** 2013-04-10

**Authors:** Falko Krause, Marvin Schulz, Ben Ripkens, Max Flöttmann, Marcus Krantz, Edda Klipp, Thomas Handorf

**Affiliations:** ^1^Theoretical Biophysics, Humboldt-Universität zu Berlin, 10115 Berlin, Germany and ^2^Codecentric AG, 40591 Düsseldorf, Germany

## Abstract

**Motivation:** The rapid accumulation of knowledge in the field of Systems Biology during the past years requires advanced, but simple-to-use, methods for the visualization of information in a structured and easily comprehensible manner.

**Results:** We have developed biographer, a web-based renderer and editor for reaction networks, which can be integrated as a library into tools dealing with network-related information. Our software enables visualizations based on the emerging standard Systems Biology Graphical Notation. It is able to import networks encoded in various formats such as SBML, SBGN-ML and jSBGN, a custom lightweight exchange format. The core package is implemented in HTML5, CSS and JavaScript and can be used within any kind of web-based project. It features interactive graph-editing tools and automatic graph layout algorithms. In addition, we provide a standalone graph editor and a web server, which contains enhanced features like web services for the import and export of models and visualizations in different formats.

**Availability:** The biographer tool can be used at and downloaded from the web page http://biographer.biologie.hu-berlin.de/. The different software packages, including a server-indepenent version as well as a web server for Windows and Linux based systems, are available at http://code.google.com/p/biographer/ under the open-source license LGPL.

**Contact:** edda.klipp@biologie.hu-berlin.de or handorf@physik.hu-berlin.de

## 1 INTRODUCTION

The web resource Pathguide (Bader *et al.*, 2006) currently lists 35 databases for ‘Pathway Diagrams’ of biochemical reaction networks, each using different means of visualization and containing at least a two digit number of network graphs. Although other scientific areas have used universal visual languages for decades, e.g. circuit diagrams in electrical engineering or UML in software engineering, Systems Biology was missing unified network visualizations until the introduction of the Systems Biology Graphical Notation (SBGN) (Le Novère *et al.*, 2009). SBGN provides standardized glyphs for the representation of cellular networks in three different languages: Activity Flow diagrams, depicting the flow of information through a network, Entity Relationship diagrams, showing possible interactions of network components, and Process Descriptions (PD), highlighting the temporal order of such interactions, all of which are fully supported by biographer.

Several tools supporting SBGN compliant visualization are already available. However, they suffer from limitations. Desktop applications like CellDesigner (Funahashi *et al.*, 2003), PathVisio (van Iersel *et al.*, 2008) and SBGN-ED (Czauderna *et al.*, 2010) require the user to install them in their operating system, creating a barrier for their initial use. Web-based visualization tools do not provide ready to use libraries (e.g. Reactome, Joshi-Tope *et al.*, 2005) and/or do not adhere to representation standards (e.g. KEGG, Ogata *et al.*, 1999). Such libraries would be of great help in the development of novel tools supporting SBGN-compliant visualizations. The libSBGN (van Iersel *et al.*, 2012) enables basic manipulations of SBGN-ML files, a text-based exchange format for SBGN graphics, but it does not provide methods for interactive visualizations.

The biographer project is trying to address these needs by providing an open source web-based SBGN renderer and editor. It includes a web service for rendering SBGN and a core library based on HTML5 that can easily be integrated into other tools and web resources. Our software has been tested within different environments and works in up-to-date browsers like Firefox and Chrome.

## 2 SIMPLE USE CASE

**Fig. 1.**
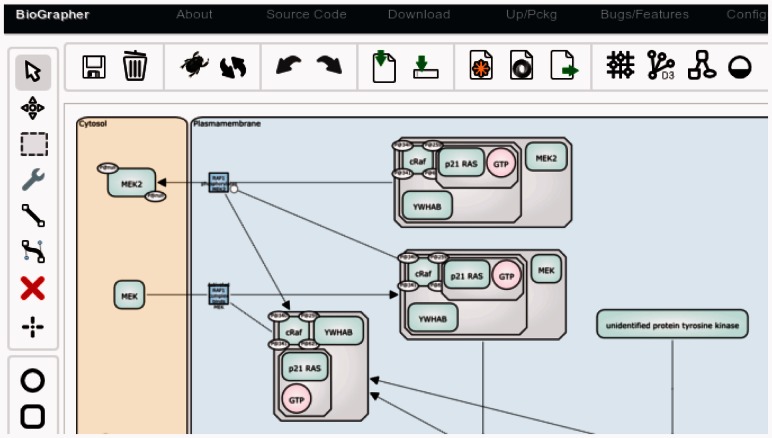
The web-based editor of biographer showing an SBGN-PD image of the MAP kinase cascade using data from the Reactome database

The simplest method to use biographer is to visit its interactive editor tools at our website (see Fig. 1). This editor allows users to create graphs from scratch by dragging glyphs onto a canvas and connecting them via arcs. Furthermore, it is possible to import existing models directly from BioModels Database, Reactome pathways or from user-provided SBML, jSBGN or SBGN-ML files. As SBML documents by default do not contain information on glyph or arc types, these are guessed from MIRIAM annotations (Le Novère *et al.*, 2005) and SBO terms (Le Novère, 2006).

After the network has been created or imported, the editor provides several tools to customize the visualization, e.g. to change the glyph color or to align and distribute nodes. Furthermore, it provides methods to layout networks using different algorithms. These algorithms include a force-directed layout provided by the d3 JavaScript library (http://d3js.org/) and two novel methods, biographer-flow and biographer-grid.

Finally, the tool allows the export of the graph in the SBGN exchange format jSBGN and in the image document formats SVG, PDF, PNG and TIFF for further editing.

## 3 USE OF BIOGRAPHER IN OTHER TOOLS

Software developers can use biographer for network visualizations on their own web sites. Given an input model in one of the aforementioned formats, our simple web service can provide SBGN compliant SVG images in an HTML Inlineframe.

The JavaScript API of biographer allows web applications to dynamically change the visualized network and its appearance. Applications using the library include our editor, semanticSBML (Krause *et al.*, 2010), the NetworkExplorer (http://cheetah.biologie.hu-berlin.de/networkexplorer/)—a tool that allows users to interactively browse through subnetworks of a large reaction network—and rxncon (Tiger *et al.*, 2012), which is able to automatically create topological jSBGN documents for reaction network in SBGN-Entity Relationship and SBGN-PD simultaneously and includes a boolean simulator with an interactive SBGN-Activity Flow visualization.

## 4 jSBGN

We have chosen to create our own JSON-based exchange format, called jSBGN, to store and transmit SBGN images. Our web services require an exchange format that is compact enough to be transmitted fast over the internet and that can be easily interpreted in scripting languages. Furthermore, jSBGN avoids the ‘no computation’ paradigm of SBGN-ML, which requires that all properties of all visible elements have to be declared in the exchanged file. For example, in jSBGN, arcs only need to provide the source and target glyph, while the start and end positions of the lines on the glyphs will be calculated. This concept makes the creation of jSBGN files simpler and lowers the barrier to integrate the biographer library into other software.

## 5 CONCLUSION

The visualization of biochemical networks, which includes metabolic, signaling and regulatory networks, has become an important task in the field of biology. In particular, with the emergence of electronic databases containing large interaction networks of biochemical species, the need for automatic or at least semi-automatic rendering of such information has increased drastically. Here, we present a tool that allows visualizing and editing of network information in web-based environments as a stand-alone application as well as a library.

Although software systems for rendering and editing SBGN images are available, biographer addresses several needs that these tools do not fulfill yet. We believe that the SBGN standard will be more widely used when its creation and usage is as simple as possible.
